# Genetic alterations in the coding region of the *bak* gene in uterine cervical carcinoma

**DOI:** 10.1038/sj.bjc.6600944

**Published:** 2003-05-13

**Authors:** K M Y Wani, N G Huilgol, T Hongyo, H Ryo K Shah, N Chatterjee, C K K Nair, T Nomura

**Affiliations:** 1 Department of Radiation Biology and Medical Genetics, Graduate School of Medicine, Osaka University, Osaka 565-0871, Japan; 2 Division of Radiation Oncology, Nanavati Hospital and Medical Research Centre, Mumbai 400056, India; 3 Biochemistry of Stress Response Section, Radiation Biology Division, BARC, Mumbai 400085, India

**Keywords:** *bak*, apoptosis, cervical cancer, mutation, radiotherapy

## Abstract

A significant frequency of mutations (six missense and one silent) was found, for the first time, at the coding region of the *bak* gene (exons 3, 4 and 6) in 42 carcinomas of the uterine cervix, while no mutations were detected in 32 non-neoplastic cervix tissues. *Bak* mutations were observed more frequently in the advanced stage and mutated cancer tissues were more resistant to radiotherapy, although trends were not statistically significant because of small sample size.

Carcinoma of the uterine cervix remains the fifth most common female neoplasm worldwide. It appears that an early event is infection by human papilloma viruses (HPV) (Lorincz *et al*, 1987). However, not all patients with HPV infection develop invasive cancer, implying that additional molecular alterations may contribute to the multistep carcinogenesis process. These alterations most probably include activation of oncogenes or inactivation of tumour suppressor genes.

The *bak* gene, a recently identified member of the Bcl-2 family of apoptosis regulatory genes, has been shown to function as a potent inducer of apoptosis (Chittenden *et al*, 1995b; Kiefer *et al*, 1995). It has been mapped to the chromosome 6 p 21.3 (Herberg *et al*, 1998). A high incidence of loss of heterozygosity was found at this region in human cervical cancer (Kersemaekers *et al*, 1998), suggesting the involvement of a tumour suppressor gene. Since transformation of the cervical epithelial tissue to carcinoma is associated with the progressive inhibition of apoptosis (Nair *et al*, 1999) and *bak*-mediated apoptosis occurs in these cells (Krajewski *et al*, 1996), abrogation of this *bak*-mediated apoptosis could lead to transformation of the cervical epithelium. The *bak* gene may be acting as a tumour suppressor gene in cervical carcinoma.

We, therefore, studied the potential role of the *bak* gene as a tumour suppressor gene in cervical carcinoma by analysing the coding region for the presence of mutations and assessed its relation to the response to radiotherapy.

## PATIENTS AND METHODS

### Cervix cancer cases and radiotherapy

Biopsied specimens from 42 patients with cervical carcinoma were obtained with informed consent prior to the initiation of radiotherapy at the Nanavati Hospital. The average age of the patients at biopsy was 51.6±3.9 years (mean ± 95% CI). Patients were classified as 12 stage II cases and 30 stage III cases according to the criteria defined by the Federation of Gynecologists and Oncologists. All patients were irradiated with ^60^Co *γ*-rays by Theratron 780C (Atomic Energy of Canada Ltd, Chalk River, ON, Canada), a telecobalt equipment. Patients were treated at a dose rate of 1.2–1.4 Gy min^−1^ with a parallel opposed field, box technique or with multiple beams to encompass the entire pelvic disease. The upper margin of the field was placed at the L5/S1 junction, whereas the lower margin included the lowest palpable disease with a centimetre of margin. All patients received 50 Gy in 5 weeks with conventional fractionation (2 Gy daily for 5 weeks with no radiation on Saturday and Sunday). A boost with brachytherapy or with reduced field of external radiation was delivered after a gap of 8–10 days of external radiation. A minimum of 70 Gy was delivered with a maximum dose of 74 Gy (Huilgol *et al*, 1988). Patients were assessed for response within a week of conclusion of irradiation. The initial response as assessed clinically was categorised as complete or partial by the WHO criterion (WHO, 1979). Briefly, complete response is defined as complete regression of clinically detectable tumour, while partial is anything less than complete response but more than 50% of the initial tumour burden (Huilgol and Chatterjee, 1996).

Serial paraffin sections (5 *μ*m thickness) were made and stained with haematoxylin and eosin. Following the microscopic view, parts of unstained paraffin sections containing tumour tissues were scraped using a sterile surgical blade under the microscope and used for DNA extraction. As controls, paraffin sections of non-neoplastic cervical tissue specimens taken from 32 patients undergoing hysterectomy at Nanavati Hospital for various non-neoplastic uterine diseases (30 chronic endocervicitis tissues and two normal cervix tissues from endometroid adenocarcinoma patients) were examined. The average age of the patients was 44.9±3.8 years (mean ±95% CI).

### Examination of *bak* mutation

**Figure 1:**
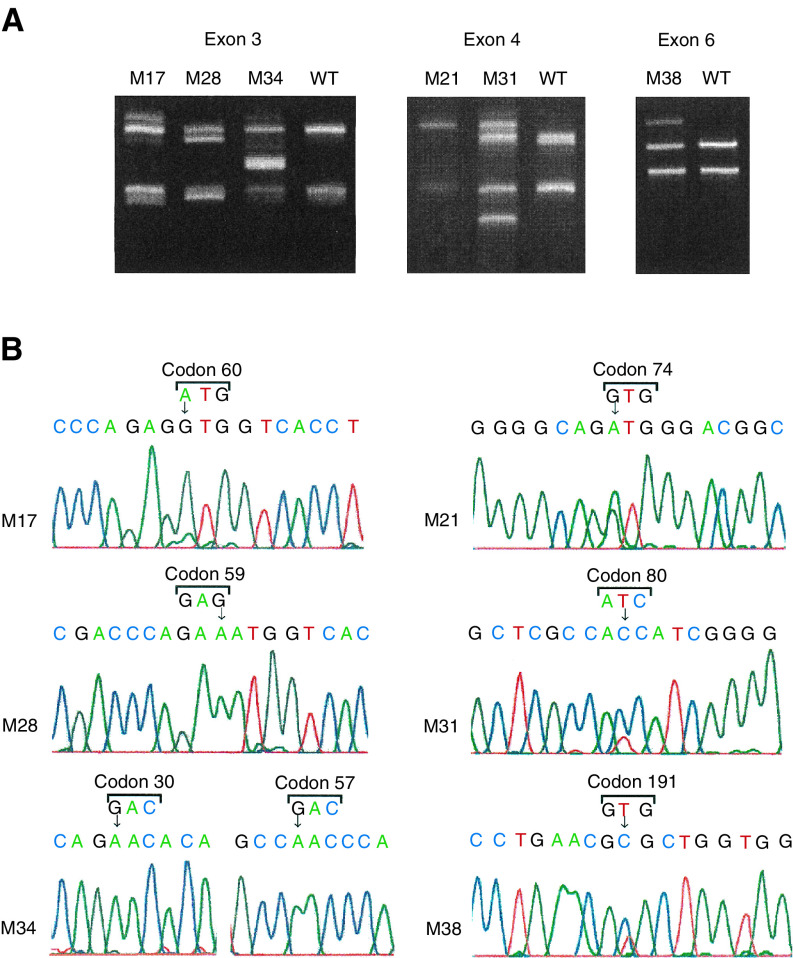
Detection of *bak* mutations in cervical carcinoma by cold-SSCP and direct sequencing. (**A**) Cold-SSCP profiles. PCR products from tumour DNA were run alongside a wild-type (WT) control (human lymphocyte DNA). Bands showing altered mobility were observed in six cervical cancer cases (M17, 28, 34 for exon 3, M21, 31 for exon 4 and M38 for exon 6). (**B**) Sequencing profiles of the bands showing altered mobility on the SSCP gel. Arrows indicate the bases altered from wild type to mutant type.

PCR amplification followed by SSCP analysis and direct sequencing was carried out to detect mutations. Briefly, DNA was extracted from microdissected paraffin-embedded sections using the DNeasy tissue kit (QIAGEN Inc., Valencia, CA, USA), and exons 3, 4 and 6 of the *bak* gene were amplified by PCR (Geneamp PCR system 9700; Perkin-Elmer, Norwalk, CA, USA). The primer pairs used for PCR amplification were 5′-TGCCTCCCTGAAGATGTCCT-3′ and 5′-TGACTCCCAGCTTTGATCCT-3′ for exon 3, 5′-GGCAGGGTATGGTATGGTTG-3′ and 5′-TCCCGACTGC-CTGGTTACTG-3′ for exon 4, and 5′-GCAAGGGAACAGAGAAGGCA-3′ and 5′-TGACCACCTTGTTTCTCCCG-3′ for exon 6 as reported previously (Kondo *et al*, 2000). PCR products were subjected to SSCP without radioisotopes (cold-SSCP) (Hongyo *et al*, 1993). The optimal temperatures for electrophoresis were 25°C for exons 3 and 6 and 20°C for exon 4. The SSCP bands, which showed altered mobility as compared to the wild-type control (human lymphocyte DNA), were extracted from the gel and reamplified by PCR to enrich the mutated alleles (Figure 1A

). The mutated alleles were purified using the QIAquick PCR purification kit (QIAGEN Inc.). Direct sequencing was performed by the dideoxy chain termination method using the Big Dye Terminator cycle sequencing kit (Perkin-Elmer) and the automated DNA sequencer (ABI prism 3100 genetic analyser; Perkin-Elmer) (Figure 1B). The same primers were used for both PCR and direct sequencing. DNA extraction, PCR–SSCP analysis and sequencing of the mutated alleles were repeated more than twice to rule out the possibility of contamination and artifacts.

Statistical analysis was carried out by the SPSS statistical system (SPSS Inc., Chicago, IL, USA).

## RESULTS AND DISCUSSION

Six transition type mutations and one silent mutation of the *bak* gene were observed in 42 cervical cancer specimens (Table 1

**Table 1:** Mutations in coding region of the *bak* gene in cervical carcinoma

				**Mutations at**		
	**Stage**	**No. of cases**	**Cases with mutation**	**exon 3**	**exon 4**	**exon 6**
Cancerous tissues	II	12	1	1^a^	0	0
	III	30	5	3^b^	2^c^	1^d^
Total	–	42	6^e^	4	2	1
Non-neoplastic tissues	–	32	0	0	0	0

a Codon 60 (ATG→GTG: Met→Val).

b Codon 30 (GAC→AAC: Asp→Asn)+codon 57 (GAC→AAC: Asp→Asn), codon 59 (GAG→GAA: Glu→Glu).

c Codon 74 (GTG→ATG: Val→Met), codon 80 (ATC→ACC: Leu→Thr).

d Codon 191 (GTG→GCG: Val→Ala).

e *P*<0.05 *vs* control by Fisher's exact test.

). Four mutations were found in exon 3 of the *bak* gene in three cases: two in exon 4 in two cases and one in exon 6 in one case. One case showed a double mutation in exon 3. No sequence alterations were found in any of the 32 non-neoplastic cervical tissues. We have, for the first time, shown a significant increase of mutations in the functional domain of the *bak* gene in human cervical carcinoma. In this study, mutations in the *bak* gene occurred more frequently in advanced-stage tumours (16.7% in stage III *vs* 8.3% in stage II), although the difference was not statistically significant. It may be a late event in cervical carcinogenesis. This is in agreement with studies of the *bak* gene in gastric and colorectal cancers where most of the mutations occurred at advanced stage (Kondo *et al*, 2000). Furthermore, cervical cancer tissues with *bak* mutations showed a decreased response to radiotherapy in comparison to those without *bak* mutation (Table 2

**Table 2:** Pre-existing *bak* mutations in stage III cervical carcinomas and their response to radiotherapy

		**Response to radiotherapy**^b^	
***bak* mutation**^a^	**No. of cases**	**Partial**	**Complete**
+	4	3^c^	1
−	26	8	18

a Missense mutations detected in the biopsied specimens taken prior to radiotherapy.

b WHO criterion (9, 10).

c *P*=0.12 by Fisher's exact test.

). However, the difference was not statistically significant because of the small sample size (*P*=0.12).

Bak, which is a member of the Bcl-2 family, shows the presence of the BH1, BH2 and BH3 homology domains and a membrane-anchoring region (Chittenden *et al*, 1995a). The *bak* gene functions by binding and inhibiting the antiapoptotic molecule Bcl-x_L_, thereby inducing apoptosis. The BH3 domain of the *bak* gene, encoded by exon 4, has been shown to be responsible for its ability to induce apoptosis as well as bind Bcl-x_L_ (Chittenden *et al*, 1995a), while constructs lacking exon 3 did not influence the cytotoxicity of *bak* in Rat-1 cells. Deletion of the membrane-anchoring region of the *bak* gene, located in exon 6, was found to reduce its cytotoxicity because of altered subcellular localisation. We have identified missense mutations in the BH3 domain in two specimens and mutation in the membrane-anchoring region in one specimen. Since these mutations occurred in domains responsible for Bcl-x_L_ binding and subcellular localisation, it is likely that these mutations resulted in loss of proapoptotic function of the *bak* gene in these specimens and caused the reduced response to radiotherapy. In two cervical cancer tissues, however, missence mutations were found in exon 3. Further study is required to know the contribution of mutations in exon 3 to cervical carcinogenesis.

The *bak* gene has been shown to induce apoptosis in a *p53*-independent manner in lung cancer cell lines (Pataer *et al*, 2000). In most cervical carcinomas, *p53* is nonfunctional either because of HPV infection or because of mutations in the *p53* gene (Lazo, 1999). In the absence of functional *p53*, *bak*-mediated apoptosis may occur in the cervical epithelium. Abrogation of this *bak*-mediated apoptosis could lead to the development of cervical carcinomas at least in a subset of cases and results in the reduced response to radiotherapy.
